# Endplate Lesions of the Lumbar Spine: Biochemistry and Genetics

**DOI:** 10.3390/genes16070738

**Published:** 2025-06-26

**Authors:** Alessandra Colombini, Vincenzo Raffo, Angela Elvira Covone, Tito Bassani, Domenico Coviello, Sabina Cauci, Ludovica Pallotta, Marco Brayda-Bruno

**Affiliations:** 1 Orthopaedic Biotechnology Lab, IRCCS Istituto Ortopedico Galeazzi, 20161 Milan, Italy; 2 Dipartimento di Medicina di Precisione e Rigenerativa e Area Jonica (Di.Me.Pre-J), Università Degli Studi di Bari, Valenzano, 70121 Bari, Italy; v.raffo@phd.uniba.it; 3 Laboratory of Human Genetics, IRCCS Istituto Giannina Gaslini, 16147 Genova, Italy; angelacovone@gaslini.org (A.E.C.); domenicocoviello@gaslini.org (D.C.); 4 Laboratory of Biological Structures Mechanics, IRCCS Istituto Ortopedico Galeazzi, 20161 Milan, Italy; tito.bassani@grupposandonato.it; 5 Department of Medicine, School of Medicine, University of Udine, 33100 Udine, Italy; sabina.cauci@uniud.it; 6 IRCCS Istituto Ortopedico Galeazzi, 20161 Milan, Italy; ludovica.pallotta@gmail.com (L.P.); marco.braydabruno@grupposandonato.it (M.B.-B.)

**Keywords:** endplate lesions, lumbar spine, genetics, biochemistry

## Abstract

Background/Objectives: Endplate lesions of the lumbar spine are often asymptomatic and frequently observed incidentally by radiological assessment. Variants in the vitamin D receptor gene (*VDR*) and an increase in some biochemical markers related to the osteo-cartilaginous metabolism were found in patients with endplate lesions. The aim of this study was to identify biochemical and genetic markers putatively associated with the presence of endplate lesions of the lumbar spine. Methods: Quantification of circulating bone remodeling proteins was obtained from 10 patients with endplate lesions and compared with age- and sex-matched controls. Whole exome sequencing (WES) was performed on patient genomic DNA using the Novaseq 6000 platform (Illumina, San Diego, CA, USA), obtaining a median read depth of 117×–200×, with ≥98% of regions covering at least 20×. The sequencing product was aligned to the reference genome (GRCh38.p13-hg38) and analyzed with Geneyx software. Results: We observed modifications in the levels of circulating proteins involved in bone remodeling and angiogenesis. We identified variants of interest in aggrecan (*ACAN*), bone morphogenetic protein 4 (*BMP4*), cytochrome P450 family 3 subfamily A member 4 (*CYP3A4*), GLI family zinc finger 2 (*GLI2*), heparan sulfate proteoglycan 2 (*HSPG2*), and mesoderm posterior bHLH transcription factor 2 (*MESP2*). *VDR* polymorphism (rs2228570) was present in nine patients, with the homozygotic ones having more severe endplate lesions and higher levels of the analyzed circulating markers in comparison with heterozygotic patients. Conclusions: These data represent interesting evidence of genetic variants, particularly in *VDR*, and altered levels of circulating markers of bone remodeling associated with endplate lesions, which should be confirmed in a larger population. The hypothesis suggested by our results is that the endplate lesions could be the consequence of an altered ossification mechanism at the vertebral level.

## 1. Introduction

Familial Scheuermann’s disease or spinal osteochondrosis is described in the rare disease portal Orphanet with the ORPHA code 3135. The clinical/radiological features of this pathology include irregularities of the vertebral endplates, subchondral sclerosis, presence of Schmorl’s nodes (upper- or lower-disc herniation into the spongious bone of the vertebral body), disc-space narrowing and vertebral wedging [1,2]. This disorder is not considered generalized per se, as the typical lesions of osteochondrosis are focal in nature. However, osteochondrosis may occur in multifocal locations in the same individual, with lesions often being bilaterally symmetrical [3].

Vertebral osteochondrosis involves defects in the cartilage endplate of vertebrae, which is devoted to the vertical growth of the vertebral body. Morphological studies of spinal osteochondrosis showed the presence of sparse disorganized fibrils in the cartilage matrix, which are likely associated with disturbed collagen synthesis and an abnormal collagen/proteoglycan ratio [2,4].

The etiology of osteochondrosis is likely multifactorial [5]. Evidence highlights the involvement of an impaired blood supply causing oxygen and nutrition insufficiency at the vertebral ossification nuclei and consequent cell necrosis, but mechanical damage/repeated microtrauma can also contribute [6].

An autosomal dominant mode of inheritance [7], with high prevalence in male monozygotic twins [8,9], supports the hypothesis of genetic etiology.

In some cases, especially when the pathology is present at the lumbar level, spine osteochondrosis is asymptomatic, and the related low back pain (LBP) appears only in adulthood [6,10].

We supposed that the degree of the lesion, from low to high, is a progressive or continuous process [11]. Recently, a study on 750 healthy Chinese subjects aged 20–60 has supported that the occurrence and development of endplate lesions is a cumulative process, showing that people with higher grades of lesions are older in both sexes and that the majority of endplate lesions are asymptomatic and frequently observed incidentally by radiological assessment [12].

An Italian cohort study performed on adult patients with LBP found that 26.8% of male patients and 8.5% of female patients had spine osteochondrosis [13,14]. Studies of the same group of researchers were dedicated to the development of a score based on the imaging features of vertebral osteochondrosis [11] and to the identification of peculiar biochemical and genetic markers putatively associated with the severity of the pathology [13,14,15,16,17]. In these studies, vertebral osteochondrosis showed peculiar genotypic and biochemical features related to vitamin D and osteo-cartilaginous metabolism. It was observed that being a male, having a high BMI and bearing specific genotypes of the vitamin D receptor gene (*VDR*) may represent risk factors for the development of higher magnetic resonance imaging (MRI) scores of endplate lesions, and that higher total MRI score directly correlates with higher levels of markers of type I and II collagen degradation [15,17]. On the contrary, a direct association between the severity of the score with more serious injuries and LBP was not found. These aspects suggest that osteochondrosis is an insidious pathology, asymptomatic at the beginning and for a long time, but provoking progressive structural damage of vertebral endplates and lasting in painful degeneration of the vertebral units.

The endplates involved in this pathology represent peculiar structures, essential to provide nutrients from the bloodstream to the avascular disc and for the molecular connection between discs and vertebrae [12].

Since osteochondrosis is acknowledged as a condition presenting degenerative-necrotic alteration of the epiphyseal and apophyseal growing ossification nuclei, the understanding of the biological mechanisms of the endochondral ossification is essential to identify the possible causes of endplate damages. In fact, the lesions related to osteochondrosis involve both the bony and cartilaginous tissues and are likely the consequence of an altered ossification mechanism at the vertebral level during skeletal growth, consisting of the replacing of the growth cartilage by bone through a sequential process of cell proliferation, extracellular matrix synthesis, cellular hypertrophy, matrix mineralization, and vascular invasion [3].

Another possible biological mechanism underlying the development of endplate lesions is strictly connected with disc degeneration. The pro-inflammatory cytokines secreted in degenerated disc tissues are known to promote osteo-progenitor differentiation and angiogenesis, which eventually activate ectopic ossification [18] with loss of joint space, subchondral sclerosis and the formation of osteophytes.

On the basis of these considerations, the aim of the present study is to identify peculiar imaging and biochemical and genetic markers putatively associated with the presence of endplate lesions of the lumbar spine.

## 2. Materials and Methods

### 2.1. Enrolment of Patients and Sample Collection

The study was approved by the local ethics committee (San Raffaele Hospital, Milan, Italy—approval number 150/int/2019). Patients were enrolled at IRCCS Istituto Ortopedico Galeazzi (Milan, Italy) after signing an informed consent form. The inclusion criteria were as follows: age over 18 years old, no previous spine surgeries, no previous diagnosis of scoliosis or other spine diseases, absence of pregnancy and absence of obesity (BMI < 30 kg/m^2^).

From the enrolled patients, 10 were selected (5 females and 5 males; age 48.9 ± 13.5; BMI 23.2 ± 2.5 kg/m^2^) for the presence of severe endplate lesions. From these patients, whole blood was obtained and used for peripheral blood mononuclear cell (PBMC) isolation and serum collection.

Data concerning circulating bone remodeling markers in 10 matched controls (5 females and 5 males, 49.6 ± 8.6; BMI 23.2 ± 1.4 kg/m^2^) were already published by our group [19] and used as a reference control in this study.

### 2.2. Patient MRI Evaluation

**Figure 1:**
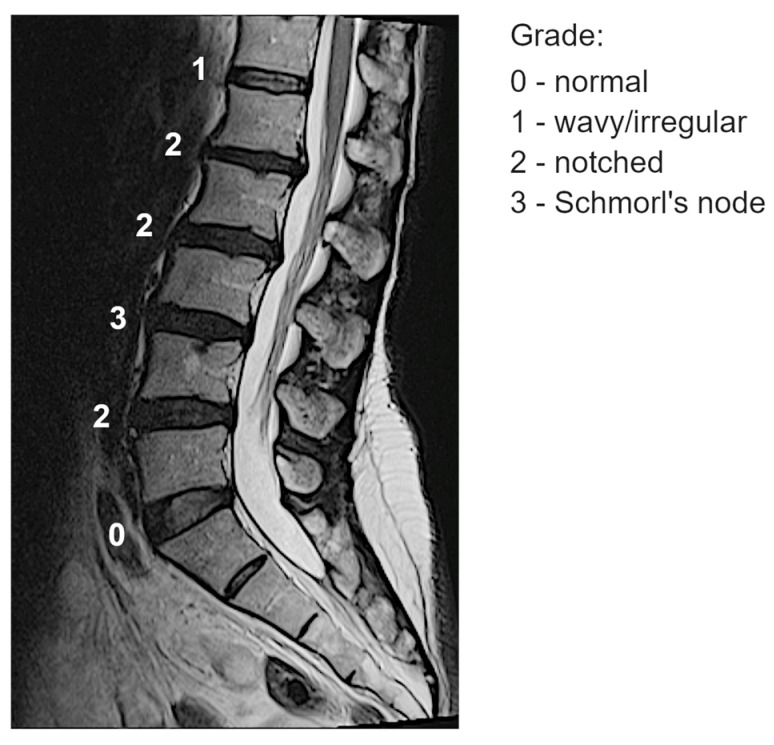
Example of classification of the endplate lesions at spinal levels from one subject at the mid-slice image.

T2-weighted sagittal MRI scans of the thoracolumbar spine were acquired using a 1.5T MR system. All images were evaluated by a single experienced spine surgeon. Endplate lesions at intervertebral levels from T12L1 to L5S1 were assessed according to a previously validated shape-based classification scheme, which demonstrated acceptable inter- and intra-observer reliability [11]. The scoring system ranges from 0 to 3, reflecting the increasing severity of endplate lesions, and is defined as follows (see an illustrative example from a single subject at the mid-slice image in Figure 1):

- Grade 0 (“normal”): No lesions are visually identified in the sagittal MRI slices encompassing the intervertebral space.
- Grade 1 (“wavy/irregular”): No specific lesions are detectable in the intervertebral space, but at least one endplate exhibits an altered shape compared to the typical curvature of a healthy intervertebral space. The endplate may appear wavy or irregular.
- Grade 2 (“notched”): A small lesion is visible in at least one sagittal MRI slice. The lesion has a V-shaped or circular appearance and is present on one or both endplates, suggesting small defects or indentations.
- Grade 3 (“Schmorl’s node”): A deep focal defect is observed in the vertebral endplate, characterized by a smooth margin and rounded appearance. Schmorl’s nodes involve disc tissue protruding through the endplate into the vertebral marrow.

The representative score at each spinal level was determined by identifying the highest severity grade observed across the image slices (e.g., if grades 0, 2, and 3 were present, the assigned score for that level was 3).

### 2.3. Protein Array of Bone Remodeling Markers

Custom Human Quantibody^®^ Array was used to determine the concentration in serum of nine circulating bone remodeling factors (RayBiotech, Norcross, GA, USA): bone morphogenic proteins (BMPs) -2, -5, -7, -9, Dickkopf-1 (Dkk-1), matrix metalloprotease (MMP)-3, platelet-derived growth factor (PDGF)-BB, transforming growth factor (TGF)β3 and tumor necrosis factor-related activation-induced cytokine (TRANCE).

### 2.4. Whole Exome Sequencing (WES)

Genomic DNA was extracted from PBMCs according to the procedure of the DNeasy Midi kit (Qiagen, Duesseldorf, Germany). WES was performed on genomic DNA using the Novaseq 6000 platform (Illumina), obtaining a median read depth between 117× and 200× with regions covered at least 20× > 98%. The sequencing product was aligned to the reference genome (GRCh38.p13-hg38), and analysis and interpretation were carried out using Geneyx software v 5.15 [20]. We identified variants of interest both by analyzing total WES data and by filtering the WES data with a dedicated gene panel and the following human phenotype ontology clinical signatures: abnormality of the vertebral endplates, abnormal form of the vertebral bodies, abnormal vertebral morphology, abnormal intervertebral disk morphology, and morbus Scheuermann.

### 2.5. Statistical Analysis

GraphPad Prism v.8.0.2 (GraphPad Software) was used for statistical analysis. Protein levels are expressed as the mean  ±  SD. The normal distribution of values was assayed by the Kolmogorov–Smirnov normality test. Unpaired comparisons between patients and controls were performed by using a two-tailed t-test. In the case of non-normally distributed values, the Mann–Whitney test was used. We set significance levels at *p* ≤ 0.05.

## 3. Results

### 3.1. Characteristics of the Enrolled Patients

All the enrolled patients were Caucasian, 3 out of 10 were overweight (BMI ≥ 25 kg/m²), and the other 7 patients were normal weight. One patient had a hyperthyroid, one had Hashimoto’s thyroiditis and one was hypertensive. All but one of the enrolled patients reported a family history of back pain. Regarding lifestyle, four patients were ex-smokers, two were non-smokers and four were smokers (one heavy). Half of the patients were doing sedentary work, while the other half were doing medium to heavy work. Furthermore, 3 of 10 patients reported vibration exposure for more than 2 h per day. Seven patients reported minimal disability, one moderate and two severe, inflicted by back pain.

### 3.2. Endplate Lesions in Patients Through MRI Score

Lesions were present at all spinal levels in almost all patients (Table 1).

Among the 60 evaluated intervertebral levels, 2 (3%) were classified as grade 0 (normal), 25 (42%) as grade 1 (wavy/irregular), 25 (42%) as grade 2 (notched), and 8 (13%) as grade 3 (Schmorl’s node), with the latter present in half of the subjects. Overall, wavy/irregular and notched lesions were observed at both upper and lower spinal levels, showing no clear association with either cranial or caudal intervertebral discs in the lumbar region. However, Schmorl’s nodes, the most severe lesion type, were absent in the most cranial upper levels from T12L1 to L1L2.

### 3.3. Circulating Marker Levels

We observed a significant increase in patients versus controls of the bone degradation marker Dkk-1 (*p* = 0.005, with 4/10 values in patients below the limit of detection vs. 9/10 values in controls), of the angiogenic marker PDGF-BB (*p* = 0.01) and of BMP-5 (*p* = 0.01). Data concerning all the evaluated markers are shown in Table 2.

### 3.4. Genetic of the Endplate Lesions

WES analysis allowed us to identify variants in the 10 selected patients. In 9 out of 10 patients, a *VDR* polymorphism (rs2228570) was observed as present in homozygosis (three patients) and heterozygosis (six patients). Furthermore, we observed different variants: three in aggrecan (*ACAN*), four in heparan sulfate proteoglycan 2 (*HSPG2*) (two in the same patient), two in bone morphogenetic protein 4 (*BMP)4* and three in GLI family zinc finger 2 (*GLI2*) (two in the same patient). Interestingly, one variant of mesoderm posterior bHLH transcription factor 2 (*MESP2*) was found in three patients, and one variant of cytochrome P450 family 3 subfamily A member 4 (*CYP3A4*) was found in two patients (Table 3).

Finally, other variants were found represented in only one patient (Supplementary Table S1) and can be grouped on the basis of the biological function of the genes. They comprise genes involved in signaling pathways such as nuclear factor kappa B subunit 1 (*NFKB1*) regulator of the NFkB complex; natriuretic peptide receptor 2 (*NPR2*), ETS variant transcription factor 2 (*ETV2*) and RUNX family transcription factor 1 (*RUNX1*), regulators of transcription; LDL receptor-related protein 4 (*LRP4*) involved in Wnt signaling and SMAD family member 3 (*SMAD3*) involved in TGFβ signaling. Other variants were found in genes related to the cellular cytoskeleton, such as filamin A (*FLNA*), myosin heavy chain 11 (*MYH11*), myosin light chain kinase (*MYLK*), nebulin (*NEB*) and thrombospondin type 1 domain containing 4 (*THSD4*). Finally, a group of variants were found in genes involved in the synthesis and remodeling of extracellular matrix components such as collagens and proteoglycans: collagen type XI α 1 chain (*COL11A1*), collagen type I α 1 chain (*COL1A1*), fibrillin 1 (*FBN1*), fibronectin 1 (*FN1*), galactosamine (N-acetyl)-6-sulfatase (*GALNS*), matrilin 3 (*MATN3*), matrix metalloprotease (*MMP2*), prolyl 3-hydroxylase 1 (*P3H1*), solute carrier family 26 member 2 (*SLC26A2*) and thrombospondin type 1 domain containing 4 (*THBS2*).

### 3.5. MRI Score and Circulating Marker Levels in Patients with VDR Variant rs2228570

The MRI score and the circulating marker levels of the cohort of nine patients presenting *VDR* variant rs2228570 were re-analyzed considering the presence of this variant in homozygosis (*n* = 3) or heterozygosis (*n* = 6). The data reported in Table 4 show that the homozygotic patients have a higher total MRI score for endplate lesions (*p* = 0.02) and higher levels of all the analyzed circulating markers, with significantly higher levels of BMP-5 (*p* = 0.05) in comparison with heterozygotic patients.

**Figure 2:**
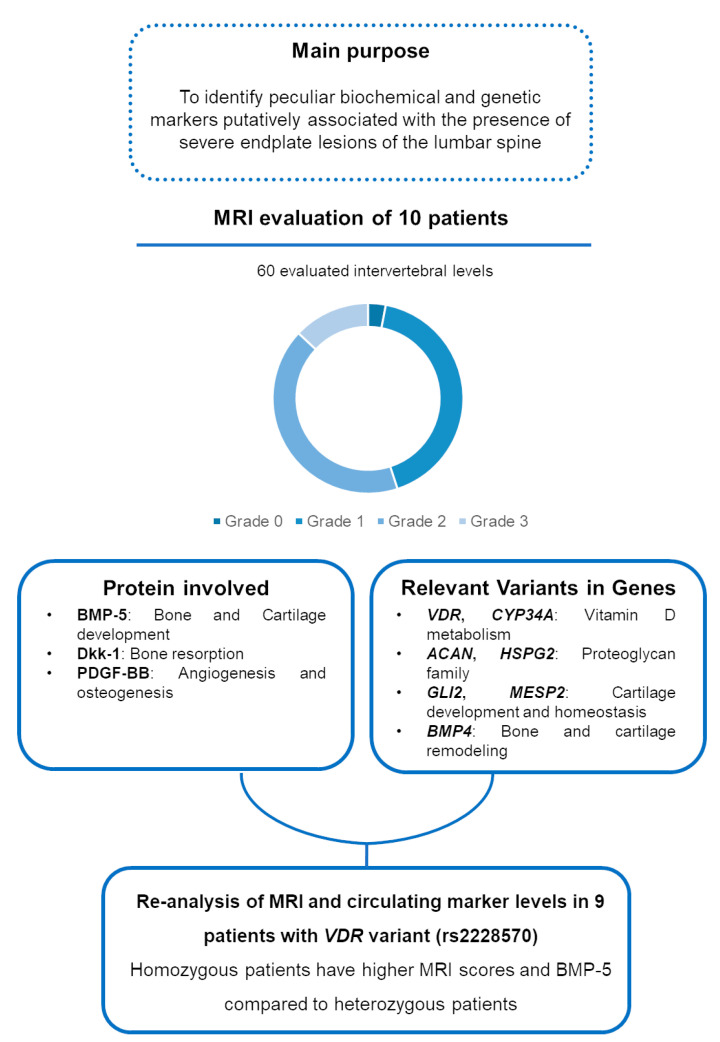
Visual diagram summarizing the main purpose and findings of the study.

Figure 2 summarizes the main findings of the study.

## 4. Discussion

The main findings of the present study are that the genetic variants identified in patients with endplate lesions support a multifactorial hypothesis for the pathogenesis of this condition and for intervertebral disc degeneration, with implications in the development, homeostasis, and metabolism of bone and cartilage.

The three proteins identified as associated with severe endplate lesions are involved in bone and cartilage development (BMP-5), in bone resorption (Dkk-1) and in angiogenesis (PDGF-BB). This is particularly relevant since the endplate defects observed in patients with osteochondrosis likely result from an altered ossification mechanism.

In particular, BMP-5 is a secreted ligand of the TGFβ superfamily of proteins that, through the activation of SMAD family transcription factors, plays a role in bone and cartilage development [21,22,23]. Another protein involved in bone remodeling is Dkk-1. It functions as an endogenous suppressor of the canonical Wnt signaling pathway [24]. Both preclinical and clinical data were suggestive that high DKK1 expression can impair osteoblast activity and cause bone loss [25].

Finally, PDGF-BB/platelet-derived growth factor receptor β signaling has a well-established role in blood vessel formation [26,27]. It correlates with the stabilization of newly formed vessels, the orchestration of cellular components for osteogenesis, and an increase in vascularity [27,28,29]. Excessive secretion of PDGF-BB from pre-osteoclasts promoted aberrant angiogenesis-dependent bone formation in subchondral bone [30,31].

In the present work, the WES analysis showed some variants from a panel of genes involved in vitamin D metabolism, osteo-cartilaginous extracellular matrix formation, cartilage development and homeostasis and endochondral ossification signaling. In a previously published paper, we observed an association between the *VDR* polymorphism rs2228570, previously named FokI, and discopathies in general and/or osteochondrosis concomitant with disc herniation [23]. In particular, the *F* allele and *FF* genotype were observed as risk factors for these conditions. In the present research, we found that this *VDR* polymorphism was present in the majority of patients (9 out of 10), and this SNP was present in homozygosis in three patients and in heterozygosis in six patients, confirming the association of this polymorphism with the presence of severe endplate defects. Interestingly, the homozygous patients showed higher MRI scores for endplate lesions and higher circulating BMP-5 levels than heterozygotes, strengthening the hypothesis that this variant is associated with the presence of more severe pathology with osteo-cartilaginous involvement. In the present work, we found two patients with a variant in *CYP3A4*, a gene potentially involved in vitamin D metabolism [32,33].

Three different variants were found in *ACAN*, encoding for a member of the aggrecan/versican proteoglycan family and four variants in *HSPG2*, and both are members of the proteoglycan family. In 3 out of 10 patients, we observed variants in these genes previously associated with spinal degeneration [34,35,36,37]. In the other two and three patients, we observed variants in *GLI2* and *MESP2*, respectively, encoding proteins involved in SHH and Notch signaling, and these genes participate in joint cartilage development and homeostasis [38,39,40,41].

In two patients, we observed a variant in *BMP4*, and the protein encoded by this gene acts through the activation of the same signaling pathway as BMP-5 and is involved in the remodeling of bone and cartilage [42].

Among the variants identified in each patient are those in *COL11A1* and *THBS2*, and these genes were already identified as potentially associated with disc-related disorders [34]. The other genes in which we observed variants in at least one patient such as *COL1A1, MATN3, GALNS, MMP2, P3H1* and *SLC2GA2* are involved in extracellular matrix remodeling and in the development and homeostasis of cartilage and bone and in collagen and proteoglycan synthesis and assembly as cartilaginous matrix components.

MRI evaluation of the enrolled patients identified the presence of endplate lesions in all subjects, with the presence of Schmorl’s nodes, the most severe types of lesions, in half of the subjects. The questionnaires allowed the collection of clinical and lifestyle-related characteristics of the enrolled patients, highlighting the presence of back pain-related disability in all subjects.

The main limitation of this study lies in the small sample size, which constrains the ability to correlate resonance scores and patient characteristics with biochemical and genetic data. Functional studies involving a larger patient cohort are necessary to better elucidate the genetic underpinnings of the disease. Additionally, the cross-sectional design of the data should be taken into account when planning future research, especially considering the potential progression of endplate lesions over time.

The strength of the study resides in the integrative approach, combining genetic analysis with biochemical and imaging evaluation to uncover a strong genotype–phenotype link. By identifying key genetic variants—particularly in the *VDR* gene—and associating them with MRI-detected endplate lesions and biochemical markers like BMP-5, the research supports a pathogenesis involving bone and cartilage metabolism.

## 5. Conclusions

Although preliminary, these findings provide compelling evidence of genetic variants—particularly in *VDR*—and altered circulating levels of cartilage and bone remodeling markers associated with endplate lesions. These results warrant confirmation in a larger patient cohort, as the endplate alterations are likely the result of disrupted ossification processes at the vertebral level.

## Figures and Tables

**Table 1:** Classification of endplate lesion types along spinal levels in evaluated subjects: grade 0 (normal), 1 (wavy/irregular), 2 (notched), and 3 (Schmorl’s node).

Subject	Spinal Level					
	T12L1	L1L2	L2L3	L3L4	L4L5	L5S1
OSTEO06	1	1	1	3	3	2
OSTEO07	2	2	3	1	2	2
OSTEO08	1	2	3	2	1	2
OSTEO13	1	2	2	2	2	1
OSTEO14	0	1	1	3	1	0
OSTEO16	2	2	2	1	1	1
OSTEO17	1	2	1	1	1	2
OSTEO23	1	1	2	2	1	1
OSTEO24	1	1	2	2	2	2
OSTEO25	2	2	3	3	1	3

**Table 2:** Circulating levels of bone remodeling markers.

		BMP-2	BMP-5	BMP-7	BMP-9	DKK-1	MMP-3	PDGF-BB	TGFb3	TRANCE
Patients (*n* = 10)	Mean ± SD (pg/mL)	6.9 ± 10.2	164.0 ± 238.8 *	112.6 ± 269.5	1.8 ± 0.5	7.7 ± 10.5 **	3445.1 ± 958.8	725.9 ± 176.9 *	44.1 ± 76.9	392.0 ± 956.5
Controls (*n* = 10)	Mean± SD (pg/mL)	3.0 ± 7.9	114.9 ± 331.1	75.5 ± 70.8	1.7 ± 1.0	0.1 ± 0.3	3699.4 ± 845.6	511.3 ± 179.3	29.0 ± 64.7	196.2 ± 304.3

BMPs-2, -5, -7, -9, Dkk-1, MMP3, PDGFBB, TGFβ3 and TRANCE. * *p* ≤ 0.05; ** *p* < 0.01.

**Table 3:** Relevant variants identified through WES analysis.

Sample ID	Gene	Inheritance	Genomic Position in hg38	Variant	Effect	Prediction	Hom/Het	NM
OSTEO06	*VDR*	AR	12:47879112-A-G	c.2T>C/p.Met1Thr	Missense	VUS	Hom	NM_000376.3
	*HSPG2*	AR	1:21828300-C-T	c.12364G>A/p.Ala4122Thr	Missense	VUS	Het	NM_005529.7
	*HSPG2*	AR	1:21854910-A-T	c.6071T>A/p.Leu2024His	Missense	VUS	Het	NM_005529.7
OSTEO08	*VDR*	AR	12:47879112-A-G	c.2T>C/p.Met1Thr	Missense	VUS	Hom	NM_000376.3
	*HSPG2*	AR	1:21880388-G-C	c.2170C>G/p.(Arg724Gly)	Missense	VUS	Het	NM_005529.7
	*BMP4*	AD	14:53949882-CCT-C	c.*148_*149delAG	3’UTR	VUS	Het	NM_001202.6
OSTEO25	*VDR*	AR	12:47879112-A-G	c.2T>C/p.Met1Thr	Missense	VUS	Hom	NM_000376.3
	*MESP2*	AR	15:89776903-AGGGCAGGGGCAGGGGCAG-AGGGCAG	c.553_564delGGGCAGGGGCAG/p.Gly185_Gln188del	Inframe indel	VUS	Het	NM_001039958.2
OSTEO13	*VDR*	AR	12:47879112-A-G	c.2T>C/p.Met1Thr	Missense	VUS	Het	NM_000376.3
	*GLI2*	AD	2:120986563-G-A	c.2191G>A/p.Gly731Arg	Missense	VUS	Het	NM_001374353.1
OSTEO14	*VDR*	AR	12:47879112-A-G	c.2T>C/p.Met1Thr	Missense	VUS	Het	NM_000376.3
	*ACAN*	AD, AR	15:88859273-G-T	c.6688G>T/p.Ala2230Ser	Missense	VUS	Het	NM_001369268.1
OSTEO16	*VDR*	AR	12:47879112-A-G	c.2T>C/p.Met1Thr	Missense	VUS	Het	NM_000376.3
	*GLI2*	AD	2:120968884-T-G	c.814T>G; p.(Ser272Ala)	Missense	VUS	Het	NM_001374353.1
	*GLI2*	AD	2:120968887-T-G	c.817T>G; p.(Tyr273Asp)	Missense	VUS	Het	NM_001374353.1
OSTEO17	*VDR*	AR	12:47879112-A-G	c.2T>C/p.Met1Thr	Missense	VUS	Het	NM_000376.3
	*ACAN*	AD, AR	15:88843433-G-A	c.836G>A/p.Arg279Gln	Missense	VUS	Het	NM_001369268.1
	*CYP3A4*	AD	7:99760901-A-G	c.1334T>C/p.Met445Thr	Missense	VUS	Het	NM_017460.6
OSTEO23	*VDR*	AR	12:47879112-A-G	c.2T>C/p.Met1Thr	Missense	VUS	Het	NM_000376.3
	*ACAN*	AD, AR	15:88878056-C-A	c.*3575C>A	Downstream	VUS	Het	NM_001369268.1
	*MESP2*	AR	15:89776903-AGGGCAGGGGCAGGGGCAG-AGGGCAG	c.553_564delGGGCAGGGGCAG/p.Gly185_Gln188del	Inframe indel	VUS	Het	NM_001039958.2
	*BMP4*	AD	14:53949883-C-CA	c.*148_*149insT	3’UTR	VUS	Het	NM_001202.6
OSTEO24	*VDR*	AR	12:47879112-A-G	c.2T>C/p.Met1Thr	Missense	VUS	Het	NM_000376.3
	*CYP3A4*	AD	7:99760901-A-G	c.1334T>C/p.Met445Thr	Missense	VUS	Het	NM_017460.6
	*HSPG2*	AR	1:21850441-C-T	c.7216G>A/p.Val2406Met	Missense	VUS	Het	NM_005529.7
	*MESP2*	AR	15:89776903-AGGGCAGGGGCAGGGGCAG-AGGGCAG	c.553_564delGGGCAGGGGCAG/p.Gly185_Gln188del	Inframe indel	VUS	Het	NM_001039958.2

AD = autosomic dominant; AR = autosomic recessive; VUS = variant of uncertain significance; Hom = homozygosis; Het = heterozygosis.

**Table 4:** MRI score and circulating marker levels in patients with *VDR* variant rs2228570 in homozygotis (Hom; *n* = 3) and heterozygotis (Het; *n* = 6) patients.

Spine Level		T12-L1	L1-L2	L2-L3	L3-L4	L4-L5	L5-S1	Total		
Hom	Mean ± SD	1.3 ± 0.6	1.7 ± 0.6	2.3 ± 1.2	2.7 ± 0.6	1.7 ± 1.2	2.3 ± 0.6	12.0 ± 1.7 *		
Het	Mean ± SD	1.0 ± 0.6	1.5 ± 0.5	1.7 ± 0.5	1.8 ± 0.8	1.3 ± 0.5	1.2 ± 0.8	8.5 ± 1.5		
Circulating markers		BMP-2	BMP-5	BMP-7	BMP-9	DKK-1	MMP-3	PDGF-BB	TGFb3	TRANCE
Hom	Mean ± SD; pg/mL	10.3 ± 13.6	401.1 ± 355.6 *	354.5 ± 448.4	2.1 ± 0.6	17.6 ± 13.9	3557.1 ± 1193.2	772.4 ± 190.6	114.0 ± 125.0	1205.5 ± 1636.4
Het	Mean ± SD; pg/mL	6.4 ± 9.8	64.5 ± 61.6	10.4 ± 12.3	1.7 ± 0.3	4.0 ± 6.1	3239.5 ± 930.6	695.9 ± 197.7	14.3 ± 14.7	50.7 ± 89.6

BMPs-2, -5, -7, -9, Dkk-1, MMP3, PDGFBB, TGFβ3 and TRANCE. * *p* ≤ 0.05.

## Data Availability

The datasets presented in this study are stored in a data repository at the following link: https://osf.io/2kehj/?view_only=593aca436fd44e2db0590410b5c2379b, accessed on 20 May 2025.

## Supplementary Materials

The following supporting information can be downloaded at: https://www.mdpi.com/article/10.3390/genes16070738/s1, Table S1: Variants found represented in only one patient.

## Abbreviations

The following abbreviations are used in this manuscript:

*VDR*	vitamin D receptor
WES	whole exome sequencing
DNA	deoxyribonucleic acid
*ACAN*	aggrecan
BMP4	bone morphogenetic protein 4
*CYP3A4*	cytochrome P450 family 3 subfamily A member 4
*GLI2*	GLI family zinc finger 2
*HSPG2*	heparan sulfate proteoglycan 2
*MESP2*	mesoderm posterior bHLH transcription factor 2
LBP	low back pain
MRI	magnetic resonance imaging
BMI	body mass index
BMP	bone morphogenic protein
Dkk-1	Dickkopf-1
MMP	matrix metalloprotease
PDGF	platelet-derived growth factor
TGF	transforming growth factor
TRANCE	tumor necrosis factor-related activation-induced cytokine
PBMC	peripheral blood mononuclear cell
SD	standard deviation
NFKB1	nuclear factor kappa B subunit 1
*NPR2*	natriuretic peptide receptor 2
*ETV2*	ETS variant transcription factor 2
*RUNX1*	RUNX family transcription factor 1
*LRP4*	LDL receptor-related protein 4
*SMAD3*	SMAD family member 3
*FLNA*	filamin A
*MYH11*	myosin heavy chain 11
*MYLK*	myosin light chain kinase
*NEB*	nebulin
*THSD4*	thrombospondin type 1 domain containing 4
*COL11A1*	collagen type XI α 1 chain
*COL1A1*	collagen type I α 1 chain
*FBN1*	fibrillin 1
*FN1*	fibronectin 1
*GALNS*	galactosamine (N-acetyl)-6-sulfatase
*MATN3*	matrilin 3
*P3H1*	prolyl 3-hydroxylase 1
*SLC26A2*	solute carrier family 26 member 2
*THBS2*	thrombospondin 2
