# Supplementary material for: Endplate Lesions of the Lumbar Spine: Biochemistry and Genetics
Source: Genes (Basel). 2025 Jun 26;16(7):738. doi: 10.3390/genes16070738 (PMC12294196; doi:10.3390/genes16070738)
Supplement: Supplementary file 1 [file genes-16-00738-s001.zip › genes-3684846-supplementary.pdf]

genes

MDPI

Table S1. Variants found represented in only one patient.

Gene	Inheritance	Genomic position in hg38	Effect	Prediction	Variant	Hom/Het
COL11A1	AD, AR	1:102979079-C-T<br>1:103031249-G-GA	Missense<br>Intron splice site region	VUS<br>VUS	c.2636G>A/p.(Arg879Gln)<br>c.652-6_652-5insT	Het
COL1A1	AD	17:50187967-C-T	Missense	VUS	c.3278G>A/p.Arg1093His	Het
ETV2	AR	19:35643385-CG-C	Del G + fs	VUS	c.348delG/p.Gly117fs*90	Het
FBN1	AD	15:48644711-T-C	Missense	VUS	c.59A>G/p.Tyr20Cys	Het
FLNA	XL	X:154358548-C-G	Missense	VUS	c.4495G>C/p.Val1499Leu	Het
FN1	AD	2:215386815-G-A	Missense	VUS	c.4486C>T/p.Arg1496Trp	Het
GALNS	AR	16:88835335-C-T	Missense	LP	c.776G>A/p.Arg259Gln	Het
LRP4	AD, AR	11:46862682-C-T	Missense	VUS	c.5309G>A/p.Arg1770Gln	Het
MATN3	AD, AR	2:20006172-C-T	Missense	LP	c.362G>A/p.Arg121Gln	Het
MMP2	AR	16:55505983-G-GT	3 UTR	VUS	c.*541_*542insT	Hom
MYH11	AD, AR	16:15747939-C-T	Missense	VUS	c.2185G>A/p.Glu729Lys	Het
MYLK	AD, AR	3:123640510-G-A	Intron splice site region	VUS	c.4620-6C>T	Het
NEB	AR	2:151501455-C-G<br>2:151692305-T-C	Missense<br>Missense	VUS<br>VUS	c.23957G>C/p.Gly7986Ala<br>c.1954A>G/p.Thr652Ala	Het in same patient
NFKB1	AD	4:102537931-A-G	Missense	VUS		Het
NPR2	AD, AR	9:35794062-C-T	Missense	VUS	c.832C>T/p.Arg278Cys	Het
P3H1	AR	1:42759261-AG-A	Del G+ fs	P	c.747delC/p.(Tyr250MetfsTer87)	Het
RUNX1	AR	21:34792310-C-G<br>21:34792315-G-GCCCCCC	Missense<br>Miss+inframe indel	VUS<br>VUS	c.1268G>C/p.(Arg423Pro)<br>c.1262_1263insGGGGGG/p.(Gly420_Gly421dup)	Het in same patient
SLC26A2	AR	5:149981490-G-A<br>5:149986273-G-GT	Missense<br>3'UTR	VUS<br>VUS	c.1897G>A/p.Asp633Asn<br>c.*4460_*4461insT	Het<br>Hom

 https://doi.org/10.3390/genes16070738

SMAD3	AD	15:67138026-C-A	5′UTR	VUS	c.207-26869C>A	Het
THBS2	AD*	6:169241794-C-T	Missense	VUS	c.859G>A/p.(Val287Met)	Het
THSD4	AD	15:71765138-G-A	Missense	VUS	c.2708G>A/p.Ser903Asn	Het
		15:71547446-G-A	Missense	VUS	c.37G>A/p.Val13Ile	Het

AD = autosomic dominant; AR = autosomic recessive; XL= X linked; P= Pathogenic; LP= Likely Pathogenic; VUS = Variant of Uncertain Significance; Hom = homozygosis; Het = heterozygosis; * Lumbar disc herniation, susceptibility to.
